# Natural Language Processing to Identify Digital Learning Tools in Postgraduate Family Medicine: Protocol for a Scoping Review

**DOI:** 10.2196/34575

**Published:** 2022-05-02

**Authors:** Hui Yan, Arya Rahgozar, Claire Sethuram, Sathya Karunananthan, Douglas Archibald, Lindsay Bradley, Ramtin Hakimjavadi, Mary Helmer-Smith, Kheira Jolin-Dahel, Tess McCutcheon, Jeffrey Puncher, Parisa Rezaiefar, Lina Shoppoff, Clare Liddy

**Affiliations:** 1 Department of Family Medicine University of Ottawa Ottawa, ON Canada; 2 Faculty of Medicine University of Ottawa Ottawa, ON Canada; 3 Bruyère Research Institute Ottawa, ON Canada; 4 Faculty of Medicine University of Toronto Toronto, ON Canada; 5 Interdisciplinary School of Health Sciences University of Ottawa Ottawa, ON Canada; 6 School of Population and Public Health University of British Columbia Vancouver, BC Canada

**Keywords:** digital learning tools, medical education, primary care, digital learning, scoping review, family medicine, bibliometric, scientometric, natural language processing, e-learning, medical curriculum, medical curricula, medical school

## Abstract

**Background:**

The COVID-19 pandemic has highlighted the growing need for digital learning tools in postgraduate family medicine training. Family medicine departments must understand and recognize the use and effectiveness of digital tools in order to integrate them into curricula and develop effective learning tools that fill gaps and meet the learning needs of trainees.

**Objective:**

This scoping review will aim to explore and organize the breadth of knowledge regarding digital learning tools in family medicine training.

**Methods:**

This scoping review follows the 6 stages of the methodological framework outlined first by Arksey and O’Malley, then refined by Levac et al, including a search of published academic literature in 6 databases (MEDLINE, ERIC, Education Source, Embase, Scopus, and Web of Science) and gray literature. Following title and abstract and full text screening, characteristics and main findings of the included studies and resources will be tabulated and summarized. Thematic analysis and natural language processing (NLP) will be conducted in parallel using a 9-step approach to identify common themes and synthesize the literature. Additionally, NLP will be employed for bibliometric and scientometric analysis of the identified literature.

**Results:**

The search strategy has been developed and launched. As of October 2021, we have completed stages 1, 2, and 3 of the scoping review. We identified 132 studies for inclusion through the academic literature search and 127 relevant studies in the gray literature search. Further refinement of the eligibility criteria and data extraction has been ongoing since September 2021.

**Conclusions:**

In this scoping review, we will identify and consolidate information and evidence related to the use and effectiveness of existing digital learning tools in postgraduate family medicine training. Our findings will improve the understanding of the current landscape of digital learning tools, which will be of great value to educators and trainees interested in using existing tools, innovators looking to design digital learning tools that meet current needs, and researchers involved in the study of digital tools.

**Trial Registration:**

OSF Registries osf.io/wju4k; https://osf.io/wju4k

**International Registered Report Identifier (IRRID):**

DERR1-10.2196/34575

## Introduction

### Background

The onset of the COVID-19 pandemic and subsequent rapid transition to distance learning have highlighted the growing need for digital learning tools [1], which include any electronic application, game, or resource that supports education [2,3]. In this study, “digital learning tools” refers to any online or offline computer-based resource, mobile app, electronic game, or resource that supports, enhances, or contributes to medical education. Students currently enrolled in postsecondary education programs are familiar with technology and eager to utilize such tools to support their education. The growing demand for these tools reflects the current preference by students for digital tools to acquire and consolidate information [4].

Digital learning tools boast a variety of benefits, including enhanced learning with fewer resources, increased levels of feedback, and more detailed assessments, making them an effective resource for learners looking to meet the challenges of medical education in a digital age [5]. Previous literature reviews have been conducted on digital learning tools in the education of health professionals [6-18]. However, little research has been done to explore what digital learning tools are currently available for postgraduate family medicine training. This scoping review will provide an overview of research activities relating to the development and use of digital learning tools in this discipline. These results could promote broader use of existing tools and help identify gaps that would inform research and development of new tools for family medicine training. The information generated from this type of review is particularly valuable in family medicine, because this field is a broad-based clinical discipline facing the unique challenges of increasing the efficiency of training, meeting increased demands for social accountability, addressing the shift toward competency-based education, and keeping up with continuous advances in medical education [19,20].

As postgraduate family medicine training evolves, it is critical to understand where and how digital learning tools are being developed, as well as how learners use and perceive them, to design validated frameworks for the development of such tools. To this end, our team is conducting a scoping review to explore, organize, and understand the breadth of knowledge regarding digital learning tools in family medicine training. To do this, we will utilize the scoping review methodology outlined by Arksey and O’Malley [21] and Levac et al [22], supplemented by natural language processing (NLP) techniques, to analyze the content and semantic structure of the included resources and perform social network analysis of their citations [23].

### Objectives

This scoping review has three major objectives: (1) identify existing digital learning tools in postgraduate family medicine training; (2) identify and compare common themes and content areas across various studies emerging from thematic analysis and NLP techniques; and (3) identify coauthorship networks in the review’s field of research to understand what resources are informing tool development.

These objectives align with the scoping review methodology. Specifically, a scoping review is useful for mapping fields with a wide and diverse range of material, and is an effective mechanism for presenting research findings to knowledge users. The NLP techniques will serve to supplement and enrich the thematic analysis, while the social network analysis will lay foundational knowledge about scientific collaboration in postgraduate family medicine digital tool research.

### Novelty

Several previous literature reviews have evaluated the use of gamification and serious games (ie, games used primarily for instruction or building skills, rather than amusement) and other types of digital learning tools in medical education [6-18]. Many of these reviews have aimed to compare specific types of digital learning tools to traditional forms of education, summarizing the findings from randomized controlled trials (RCTs). The current scoping review will identify and consolidate information about all digital learning tools, including serious games, web-based resources, mobile apps, and social media platforms. This review will also include all publication types and gray literature. Since many new tools have not yet undergone formal evaluation processes through RCTs, a search of studies beyond RCTs is vital to capturing a complete picture of available tools and evidence related to their development, implementation, and use. Furthermore, this study will focus specifically on tools used in postgraduate family medicine education and identify gaps in the development and use of digital learning tools in this broad-based area of medical training.

Additionally, high levels of heterogeneity found in other studies that examined specific disciplines or specific digital tools suggest the need for a scoping review in order to describe and classify the types of available digital learning tools, identify key concepts and definitions in the literature, and map various types of evidence [8,11,12].

Finally, our scoping review will utilize artificial intelligence to organize the structure and content of the identified literature in novel ways. NLP is a type of artificial intelligence that uses machine learning algorithms to process large volumes of text effectively and is used in semantic analysis, machine understanding, clustering, and classification [24]. Previous studies have utilized NLP to reduce the burden of the literature review process by automating the identification and selection of latent topics in papers [25-29]. Such studies have used clustering methods to organize literature by similar topics and to describe and group research activities into common themes to complement classification performed by humans [26]. As NLP develops, it may play an increasingly important role in accelerating and enhancing literature reviews.

In this study, we will use NLP techniques to assist with and supplement the data synthesis phase of the scoping review, specifically to identify common themes and content areas across various studies. Additionally, we will perform social network analysis—a technique that has been applied in diverse fields, including medical parasitology, information science, and information visualization [30-32]. We will use this analysis to examine information from chosen texts and resources to identify coauthorship and collaboration networks in the research and development of digital tools in family medicine training. By using these computational and NLP techniques, we will be able to identify major research topics and concepts and strategically recognize future directions of research and development in family medicine training.

The scoping review methodology, supported by NLP techniques, will allow us to identify and consolidate information related to existing digital learning tools in postgraduate family medicine training. This paper describes the process our team will take to identify relevant literature and collaborative networks that can be leveraged in future initiatives to design and implement digital learning tools in postgraduate family medicine training.

## Methods

### Ethics Approval

This scoping review does not involve human participants and, as such, does not require ethics approval according to the Ottawa Health Science Network Research Ethics Board. The study was registered with the OSF Registries (osf.io/wju4k).

### Design

Our approach is informed by Arksey and O’Malley’s [21] methodological framework for conducting scoping reviews, which has subsequently been enhanced by Levac et al [22]. This approach facilitates a systematic process for developing a research question, searching academic databases, screening results from these searches, extracting data from relevant studies, and collating the results for dissemination. We will engage and involve stakeholders throughout the entire project, as evidence suggests that public engagement can enhance reviews and make the results more useful [22,33]. We will adhere to the Preferred Reporting Items for Systematic Reviews and Meta-Analyses Extension for Scoping Reviews (PRISMA-ScR) guidelines [34]. Upon completing the selection of relevant articles and sources of evidence, the proposed NLP implementation will commence.

### Stage 1: Identifying the Research Question

The following research question was developed through an iterative process involving discussions with the research team and knowledge users, including clinicians, medical educators, digital learning tool developers, and students: What digital learning tools exist for postgraduate family medicine training? The study development process was informed by both the lived experience of knowledge users and findings from a preliminary nonsystematic search of the literature conducted in the summer of 2020. The nonsystematic search aimed to find evidence on digital learning tools being used in postgraduate family medicine training. Due to the heterogeneity of the literature, we concluded that a scoping review would be necessary to understand what tools were being used.

### Stage 2: Identifying Relevant Studies

The search strategy was developed in consultation with knowledge users and a health sciences librarian at the University of Ottawa.

#### Academic Literature Search

We conducted a search of 6 academic databases, including MEDLINE, ERIC (Education Resources Information Centre), Education Source, Embase, Scopus, and Web of Science, to identify literature that describes the use of digital learning tools in postgraduate family medicine training. The major concepts that defined subject heading terms and keywords were “family medicine training” and “digital learning tools” (Multimedia Appendix 1). MEDLINE, ERIC, Education Source, and Embase were searched using subject heading terms and keywords. Scopus and Web of Science were searched using only keywords, as these databases do not use subject headings. The search was built in MEDLINE and was then translated to be run in the other databases (Multimedia Appendix 2). The results of the academic literature searches were imported into Covidence software for deduplication and screening.

#### Gray Literature Search

The gray literature was searched with Google (Google LLC) to identify resources from university program websites, medical forums, and conference websites, in addition to searching for theses and dissertations. We used keywords identified in the academic literature search for the gray literature search (Multimedia Appendix 3). The search was limited to the first 10 pages of results. An advanced Google search was also used to identify relevant resources from university websites, family medicine organizations, medical school and residency organizations, and relevant conferences. The advanced search was also limited to the first 10 pages of results. The reference lists of the included articles were reviewed for additional literature relevant to our study. We did not review the reference lists of the articles found by searching the reference lists.

### Stage 3: Selecting Studies

The third stage of the scoping review was study selection, which included an initial title and abstract screening, followed by full text screening.

#### Inclusion and Exclusion Criteria

Studies were considered for inclusion if they described the design, development, implementation, or evaluation of any type of digital learning tool used for postgraduate family medicine training. We included studies of all publication types and from all countries. We excluded articles that were not written in English or French and that were published before 2010. The year 2010 was chosen as a limit because we are interested in existing or emerging technologies such as virtual reality and artificial intelligence that are presently being used in family medicine education. Given the rapid and continuous advancements in the use of technology in education, evaluations conducted before 2010 would not provide a strong indication of current technology. Moreover, a systematic review of virtual reality for the education of health professionals identified only 1 reference published before 2010 [17]. Thus, we do not expect that this choice will lead us to exclude many resources.

Since there exists a gap in the current literature examining the landscape of digital learning tools for postgraduate family medicine education, we decided to use a broad search strategy with limited exclusion criteria. However, this is an iterative process, and as such, more specific exclusion criteria will be discussed and added as we familiarize ourselves with the literature.

#### Title and Abstract Screening (Academic Literature)

Independent screening of the title and abstract of each article was performed by 2 reviewers based on the inclusion and exclusion criteria. If either reviewer included an article, it underwent full text screening. Additionally, if eligibility was unclear based on the information in the abstract, the article underwent full text screening.

#### Full Text Screening (Academic Literature)

**Figure 1:**
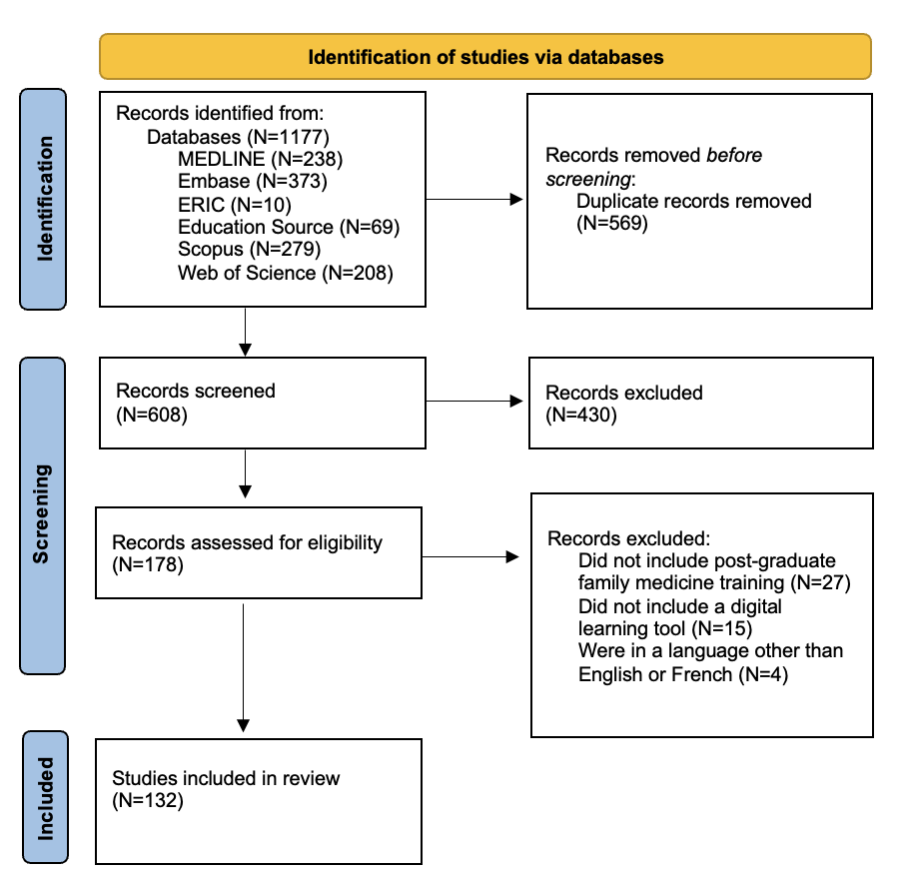
PRISMA-ScR (Preferred Reporting Items for Systematic Reviews and Meta-Analyses Extension for Scoping Reviews) flowchart.

Independent screening of each of the full texts identified for inclusion was performed by 2 reviewers, who discussed any disagreements. If an agreement could not be reached, a third person was consulted. The reasons for excluding studies were documented. A PRISMA-ScR flowchart that outlines the search decision process and the number of studies included at each phase of the process has been prepared (Figure 1) and will be disseminated in the paper describing the completed review.

#### Gray Literature Screening

Gray literature was screened by 1 reviewer. Relevant articles and resources were recorded.

### Stage 4: Charting the Data

Data will be independently extracted by 2 reviewers from the included articles and input into a data charting form. Data will include (as applicable) the title, author or authors, publication year, study objective, study design, country, description of the digital learning tool or tools, intervention description, study population, outcome measure or measures, and main findings. Charting is an iterative process, and therefore the data charting table may evolve as we familiarize ourselves with the literature. Data extraction elements may be further refined by our stakeholders, including knowledge users.

### Stage 5: Collating and Reporting the Results

Characteristics and findings from all included literature will be tabulated and summarized. Aggregate data will also be presented. We will conduct a manual thematic analysis of the included studies to highlight key themes emerging from the literature.

In parallel with the manual data extraction and analysis processes, NLP techniques will be used to process the text corpus and identify common themes in selected articles. The NLP experiments will follow a 9-step process (Textbox 1) and will be grounded in several key approaches and techniques. This is intended as an approach to supporting manual data analysis.

Rahgozar and Inkpen [35,36] have shown that NLP algorithms, such as text clustering and classification, can produce useful results from less than 500 documents with an average of 10 lines each. This supports the feasibility of conducting NLP analyses on a reduced volume of texts after the selection of relevant articles and sources of evidence has been completed. A postdoctoral fellow with experience in applying machine learning in family medicine research has designed and will perform all analyses. Textbox 1 shows the sequence of steps that will be used, and we will henceforth refer to it to when describing our NLP procedure. First, the included articles will be organized (ie, corpus development) and will undergo text preprocessing (ie, tokenization, removing stop words, and clearing images) to facilitate subsequent NLP experiments (steps 1 to 2). With the text corpus prepared, we will apply NLP to organize common language and themes and conduct a social network analysis using bibliometric and scientometric methods to visualize citation networks that emerge from the selected texts. We will develop the NLP methodology iteratively to adapt it for the task at hand and decide on the model that offers the top performance (the “champion” model) based on various evaluation indices. We will also leverage NLP to visualize relevant information and findings that will inform and facilitate the synthesis of the material.

**Step-by-step description of natural language processing.**

1. Extraction and organization of included articles (ie, corpus development)
2. Text preprocessing, including tokenization, stop word removal, and image removal
3. Data transformation and vectorization
4. Loading of the data to make it available for reusability and machine learning experimentation
5. Clustering (k-means) and evaluation
6. Latent Dirichlet allocation modeling and evaluation
7. Latent Dirichlet allocation model visualization
8. Information extraction
  Entity recognition
  Identification of top frequent terms
9. Network analysis
  Data structures, bibliographic metadata management, and data transformation
  Network visualization of citations, coauthorships, and term co-occurrences
  Node top “cardinalities” and “centralities” measurement

#### Clustering, Topic Modeling, and Information Extraction

To analyze content from the identified texts, we will use NLP techniques such as clustering, topic modeling, and information extraction (ie, the extraction of elements such as frequent terms or collocations) to conduct a more granular analysis of concepts and organize a knowledge graph in more detail [37].

Probabilistic models in machine learning help segment data based on their semantic similarities. Semantically effective representations such as bag-of-words and term-frequencies-inverse-document-frequencies will be used to transform text into vector space, allowing for traditional machine learning algorithms to process them (steps 3 to 4). We will use latent Dirichlet allocation (LDA) topic modeling for content analysis and clustering (steps 5 to 6) [37]. LDA is a probabilistic clustering model that generates latent and important topics in the documents using semantic weights. We will extend the LDA to visualize the topic terms within each cluster (step 7) [38]. The objective of clustering the corpus is to group together semantically similar contexts in a basket and extract relevant and important terms that associate together to form the main topics latent in the text. For entity recognition, an activity that involves processing a text and identifying certain occurrences of important words or expressions as belonging to particular topics of interest, we will use SpaCY, an industrial-grade, off-the-shelf model with state-of-the-art evaluation techniques to identify the most frequent names in the text (step 8) [39].

In the absence of labeled data, evaluation of the clustering methods will be based on the semantic attributes of “similar” text, measured by indices such as coherence and mutual information [40]. Other clustering algorithms, such as k-means, can also be used to decide the optimal number of clusters and the champion model using evaluation metrics such as the coherence, silhouette (a cluster validity measure that optimizes the betweenness within the densest clusters so that the furthest clusters contain the closest points possible) and elbow methods. Using the coherence and elbow methods, we will evaluate the quality of our clustering algorithms [41]. We will also evaluate how different clustering methods correspond with the subtopics and the titles of the paper groups using the Cohen κ score. As an example, we may derive clusters that illustrate how digital tools in family medicine residency education are (1) influencing educational content, (2) affecting education governance, or (3) inducing innovations in family medicine education. These insights can then be overlaid with the time dimension to observe directions, gaps, and emerging interests.

Results from these analyses will be compared to findings from the manual thematic analysis to identify similarities and differences between the 2 approaches and may suggest strengths, limitations, and opportunities for applying NLP to the data synthesis phase of scoping reviews. For example, this process may help rectify some of the challenges associated with literature reviews, such as heterogeneity in classifying research themes and maintaining a reliable balance between coverage and focus [42].

#### Bibliometric and Scientometric Methods

Using social network analysis and relevant indices, such as cardinality and centrality of nodes, we will explore the evolution and emergence of research on digital learning tools by studying the patterns and connections between authors, fields, and journals during the review study period (step 9) [43].

We will perform a social network analysis of the included citations by extracting meta information from the digital library of included articles and construct bibliographic data in standard formats. This will allow for subsequent visualization of citation networks using open-source graph visualization tools [44]. Coauthorship networks can depict scholarly teamwork and the main players given different thresholds (ie, at least 2 articles), providing insights into research trends and activities and their structures [43,45,46]. Another insightful network will be keyword co-occurrence, in which the size of the nodes will indicate the frequencies of terms and subject headings in the literature corpus. Lastly, a citation network will be produced given a threshold of at least “k” citations (k will be decided as per the norm reference sizes in the literature).

### Stage 6: Patient and Public Involvement

This scoping review was co-designed by a multidisciplinary team using an integrated knowledge translation approach. Stakeholders and knowledge users, including clinicians, medical educators, digital learning tool developers, researchers, and students, will contribute to all stages of the study. Team members assisted in developing the research question, defining the scope of the search strategy, and identifying relevant data extraction elements. They also assisted in developing a methodology for the gray literature advanced site search by identifying websites and organizations that may contain relevant information. Some stakeholder group members will participate in screening and data extraction, and all group members will be invited to contribute to the data analysis, interpretation of the results, and preparation of findings for dissemination.

## Results

As of October 2021, we have completed stages 1, 2, and 3 of the scoping review. We identified 132 studies for inclusion through the academic literature search and 127 relevant studies in the gray literature search (Figure 1). Further refinement of the eligibility criteria and data extraction has been ongoing since September 2021 (stage 4). Collation of the results (stage 5) and preparation for dissemination (stage 6) are expected to occur between September 2021 and March 2022.

## Discussion

### Overview

In this scoping review, we will identify and consolidate information and evidence related to the use of existing digital learning tools in postgraduate family medicine training. Based on the preliminary results of this review, we hypothesize that our findings will demonstrate heterogeneity in the types and diversity of tools being used. Additionally, this scoping review will lay a foundation for exploring the effective evaluation of tools as part of future research.

### The Use of NLP in Scoping Review Methodology

Although our protocol is based on established methodology [21-23], our application of NLP techniques is novel. These NLP techniques may uncover influential authors or publications and popular themes in publishing practices, which will provide important information for future literature reviews and serve as helpful context for interested newcomers in this field of research.

The breadth of literature regarding the use of digital learning tools is vast. Previous systematic reviews have identified high levels of heterogeneity in the types of digital learning tools used, measures of effectiveness, and main findings [8,10-14,18]. As such, NLP techniques may allow us to begin understanding patterns in the emergence of this topic in the literature and structuring or classifying the diverse types of digital learning tools that have been described, among other insights. Using NLP techniques such as clustering, topic modeling, and information extraction will allow us to organize common themes, content areas, and concepts between texts. This may provide a more robust thematic analysis and represents an opportunity to compare findings from traditional human-developed analysis with those identified by computational and NLP techniques [26]. Using computational techniques will provide the opportunity to explore how these techniques may be leveraged in the methodology of scoping reviews [26].

Furthermore, the application of NLP may be particularly well suited for the present review of digital learning tools in medical education, given that it is an emerging area of research with key terms that are not yet supported by well-indexed, comprehensive bibliographic databases [47]. The use of supplemental computational techniques, such as calculating cardinality and centrality of the articles based on a network model of references, will help us identify and measure the important position of the concepts within the body of knowledge. It will support traditional researcher-driven review strategies and be helpful for describing and understanding this vast and growing body of literature on digital learning tools.

Additionally, social network analysis to examine coauthorship networks has been previously applied in medical, health care, and medical education research with the aim of promoting or strengthening research collaboration [46,48-50]. Therefore, generating an understanding of the nature of collaboration in digital learning for the medical education research community may accelerate cooperative research initiatives by connecting leaders and innovators across various disciplines. Given that the development of digital learning tools is inherently an interdisciplinary pursuit, such coauthorship network analysis will be an important step in driving innovation in this field. Finally, the methods we propose to describe and group research studies are novel, and to our knowledge, have not been explored in medical education research. The utility of automating the data extraction and descriptive phases of scoping reviews through NLP depends on the nature of the dataset (ie, the selected articles) and the information sought (ie, the review question). Thus, this study represents an opportunity to establish the feasibility of these techniques in this context and produce significant foundational knowledge to support the utilization of these powerful techniques in literature reviews in the rapidly growing area of medical education research and its related disciplines.

### Limitations

Development of this protocol for our review serves to provide a detailed structure for the scoping review and to improve the transparency of the research. However, our study has several limitations. Since the objective of the review is to identify digital learning tools currently being used, we will not provide an evaluation of the quality of the digital learning tools. Additionally, digital learning tools that are not described in the academic and gray literature will not be captured in this scoping review. Any deviation from the scoping review protocol described here will be outlined in the final manuscript, accompanied by a rationale for the change.

### Dissemination Plan

The findings from this scoping review will be presented to an interdisciplinary team at the University of Ottawa’s Department of Family Medicine in order to inform the department on the current landscape of digital learning tools and aid the development of new and effective digital tools, with the aim of eventually designing digital tools for the department. As an institution that prioritizes innovation, the Department of Family Medicine actively collaborates with engineering departments and engages in co-design to develop adaptive and intelligent digital tools for education. The completion of this study, with its novel scoping review protocol, will involve continuous collaboration and effective knowledge translation among an interdisciplinary group of researchers. This interdisciplinary environment is key to enabling the exploration of novel applications of NLP in medical education and research, and it will foster further collaboration to drive innovation at the intersection of medicine and artificial intelligence. We plan to share consolidated findings in an article that will be submitted for publication in a peer-reviewed journal. Finally, findings will be disseminated through academic platforms, such as conference presentations and meetings, which will not only inform the collaborative development of digital tools to be integrated into medical curricula, but also provide an exciting, innovative, and novel framework for the application of NLP methods in medical education research. We hope that this information is of great value to educators and trainees interested in using existing tools, innovators looking to design digital learning tools that meet current needs, and researchers involved in the study of digital tools.

## Appendix

Multimedia Appendix 1 Academic literature search terms.

Multimedia Appendix 2 Search Strategy for Academic Databases.

Multimedia Appendix 3 Search strategy for Google search engine.

## Abbreviations

LDA: latent Dirichlet allocation
NLP: natural language processing
RCT: randomized controlled trial
