# Correction: Natural Language Processing to Identify Digital Learning Tools in Postgraduate Family Medicine: Protocol for a Scoping Review

**DOI:** 10.2196/40454

**Published:** 2022-06-24

**Authors:** Hui Yan, Arya Rahgozar, Claire Sethuram, Sathya Karunananthan, Douglas Archibald, Lindsay Bradley, Ramtin Hakimjavadi, Mary Helmer-Smith, Kheira Jolin-Dahel, Tess McCutcheon, Jeffrey Puncher, Parisa Rezaiefar, Lina Shoppoff, Clare Liddy

**Affiliations:** 1 Department of Family Medicine University of Ottawa Ottawa, ON Canada; 2 Faculty of Medicine University of Ottawa Ottawa, ON Canada; 3 Bruyère Research Institute Ottawa, ON Canada; 4 Faculty of Medicine University of Toronto Toronto, ON Canada; 5 Interdisciplinary School of Health Sciences University of Ottawa Ottawa, ON Canada; 6 School of Population and Public Health University of British Columbia Vancouver, BC Canada

In “Natural Language Processing to Identify Digital Learning Tools in Postgraduate Family Medicine: Protocol for a Scoping Review” (JMIR Res Protoc 2022;11(5):e34575) the authors noted two errors.

1. In the *Abstract* of the originally published article, the trial registration details did not appear correctly. These details have been corrected as follows:

OSF Registries osf.io/wju4k; https://osf.io/wju4k

2. In the originally published *Ethics Approval* section under *Methods*, the statement regarding trial registration appeared as follows:

The study was registered with the Open Science Framework (0.17605/OSF.IO/WJU4K).

It has been corrected as follows:

The study was registered with the OSF Registries (osf.io/wju4k).

The correction will appear in the online version of the paper on the JMIR Publications website on June 24, 2022, together with the publication of this correction notice. Because this was made after submission to PubMed, PubMed Central, and other full-text repositories, the corrected article has also been resubmitted to those repositories.

