# Supplementary material for: Natural Language Processing to Identify Digital Learning Tools in Postgraduate Family Medicine: Protocol for a Scoping Review
Source: JMIR Res Protoc. 2022 May 2;11(5):e34575. doi: 10.2196/34575 (PMC9112078; doi:10.2196/34575)
Supplement: Multimedia Appendix 1 [file resprot_v11i5e34575_app1.docx]

**Appendix A: Academic Literature Search Terms**

**Subject Headings**

In addition to searching the text for keywords, we searched the database’s catalog of subject headings/concepts with the below subject heading terms. The databases were searched for articles that fall under these subject headings.

**Table A1. Subject headings for search in MEDLINE**

| **Family Medicine Training** | **Digital Learning Tools** |
| --- | --- |
|  | Computer-Assisted Instruction |
|  | Microcomputers |
|  | Computers, Handheld |
|  | Smartphone |
|  | Smart Glasses |
|  | Minicomputers |
|  | Simulation Training |
|  | High Fidelity Simulation Training |
|  | Video Games |
|  | Mobile Applications |
|  | Virtual Reality |
|  | Social Media |
|  | Augmented reality |

**Keywords**

The databases were searched for articles that contain the below keywords in either the title or abstract.

Search syntax notes:

* = truncation

Truncation allows one to search for a word / phrase that may have multiple endings. For example, *residen** would find articles with the terms *resident*, *residents*, *residency*, etc.

? = wildcard (optional character)

The optional character wildcard character may represent any character or no character. For example, *web?based* would find articles with the terms *web-based* and *web based*.

adj# = adjacency

Adjacency syntax allows one to identify when two terms are within a given number of words of one another. For example, *family medicine adj2 training* would find articles with the terms *family medicine training*, *family medicine resident training*, *training for family medicine*, etc.

**Table A2. Keywords used to search academic databases**

| **Family Medicine Training** † | | **Digital Learning Tools** |
| --- | --- | --- |
| **Family Medicine** | **Training** |  |
| family medic* | student* | (digital adj1 learn*) |
| primary care | graduate* | digital resource* |
| family physician* | clerk* | web?based​ |
| family doctor* | fellow* | game?based |
| general practi* | intern* | digital tool* |
|  | residen* | gamification​ |
|  | educat* | serious gam*​ |
|  | train* | game |
|  | post?graduate* | games |
|  | tutor* | gaming |
|  |  | learning module​* |
|  |  | e?learning​ |
|  |  | (virtual adj1 learning) |
|  |  | (distance adj1 education​) |
|  |  | (online adj1 learning) |
|  |  | (computer?assisted adj1 instruction) |
|  |  | (digital adj1 education) |
|  |  | mobile app* |
|  |  | virtual reality |
|  |  | augmented reality |
|  |  | computer simulation |
|  |  | smartphone |
|  |  | tablet |
|  |  | social media |
|  |  | Twitter |
|  |  | Facebook |
|  |  | Instagram |
|  |  | TikTok |
|  |  | WhatsApp |

† Each keyword in *Family Medicine* sub-column will be combined with each keyword in the *Training* sub-column with adjacency syntax to create keywords under the *Family Medicine Training* concept.

**Appendix B: Search Strategy for Academic Databases**

**Table B1. Search strategy for MEDLINE-Ovid**

| **Search** | **Search term** |
| --- | --- |
| 1 | ((family medic* or primary care or family physician* or family doctor* or general practi*) adj3 (student* or graduate* or clerk* or fellow* or intern* or residen* or educat* or train* or post?graduate* or tutor*)).ti,ab,kf. |
| 2 | Computer-Assisted Instruction/ |
| 3 | exp microcomputers/ or minicomputers/ |
| 4 | simulation training/ or high fidelity simulation training/ |
| 5 | Video Games/ |
| 6 | Mobile Applications/ |
| 7 | virtual reality/ |
| 8 | social media/ |
| 9 | augmented reality/ |
| 10 | ((digital adj1 learn*) or digital resource* or web?based or game?based or digital tool* or gamification or serious gam* or game or games or gaming or learning module* or online module* or e?learning or (virtual adj1 learning) or (distance adj1 education) or (online adj1 learning) or (computer?assisted adj1 instruction) or (digital adj1 education) or mobile app* or virtual reality or augmented reality or computer simulation or smartphone or tablet or social media or Twitter or Facebook or Instagram or TikTok or WhatsApp).ti,ab,kf. |
| 11 | 2 or 3 or 4 or 5 or 6 or 7 or 8 or 9 or 10 |
| 12 | 1 and 11 |
| 13 | limit 12 to yr="2010 -Current" |

**Appendix C: Search Strategy for Google Search Engine**

((family medic* OR primary care OR family physician* OR family doctor* OR general practi*) AND (student* OR graduate* OR clerk* OR fellow* OR intern* OR residen* OR educat* OR train* OR post-graduate* OR postgraduate* OR post graduate* OR tutor*)) AND ((digital AND learn*) OR digital resource* OR web based OR web-based OR game based OR game-based OR digital tool* OR gamification OR serious gam* OR game OR games OR gaming OR learning module* OR online module* OR e-learning OR elearning OR (virtual AND learning) OR (distance AND education) OR (online AND learning) OR (computer-assisted AND instruction) OR (computer assisted AND instruction) OR (digital AND education) OR mobile app* OR virtual reality OR augmented reality OR computer simulation OR smartphone OR tablet OR social media OR Twitter OR Facebook OR Instagram OR TikTok OR WhatsApp)
