# Supplementary material for: Natural Language Processing to Identify Digital Learning Tools in Postgraduate Family Medicine: Protocol for a Scoping Review
Source: JMIR Res Protoc. 2022 May 2;11(5):e34575. doi: 10.2196/34575 (PMC9112078; doi:10.2196/34575)
Supplement: Multimedia Appendix 2 [file resprot_v11i5e34575_app2.docx]

**Appendix B: Search Strategy for Academic Databases**

**Table B1. Search strategy for MEDLINE-Ovid**

| **Search** | **Search term** |
| --- | --- |
| 1 | ((family medic* or primary care or family physician* or family doctor* or general practi*) adj3 (student* or graduate* or clerk* or fellow* or intern* or residen* or educat* or train* or post?graduate* or tutor*)).ti,ab,kf. |
| 2 | Computer-Assisted Instruction/ |
| 3 | exp microcomputers/ or minicomputers/ |
| 4 | simulation training/ or high fidelity simulation training/ |
| 5 | Video Games/ |
| 6 | Mobile Applications/ |
| 7 | virtual reality/ |
| 8 | social media/ |
| 9 | augmented reality/ |
| 10 | ((digital adj1 learn*) or digital resource* or web?based or game?based or digital tool* or gamification or serious gam* or game or games or gaming or learning module* or online module* or e?learning or (virtual adj1 learning) or (distance adj1 education) or (online adj1 learning) or (computer?assisted adj1 instruction) or (digital adj1 education) or mobile app* or virtual reality or augmented reality or computer simulation or smartphone or tablet or social media or Twitter or Facebook or Instagram or TikTok or WhatsApp).ti,ab,kf. |
| 11 | 2 or 3 or 4 or 5 or 6 or 7 or 8 or 9 or 10 |
| 12 | 1 and 11 |
| 13 | limit 12 to yr="2010 -Current" |
