# Supplementary material for: Natural Language Processing to Identify Digital Learning Tools in Postgraduate Family Medicine: Protocol for a Scoping Review
Source: JMIR Res Protoc. 2022 May 2;11(5):e34575. doi: 10.2196/34575 (PMC9112078; doi:10.2196/34575)
Supplement: Multimedia Appendix 3 [file resprot_v11i5e34575_app3.docx]

**Appendix C: Search Strategy for Google Search Engine**

((family medic* OR primary care OR family physician* OR family doctor* OR general practi*) AND (student* OR graduate* OR clerk* OR fellow* OR intern* OR residen* OR educat* OR train* OR post-graduate* OR postgraduate* OR post graduate* OR tutor*)) AND ((digital AND learn*) OR digital resource* OR web based OR web-based OR game based OR game-based OR digital tool* OR gamification OR serious gam* OR game OR games OR gaming OR learning module* OR online module* OR e-learning OR elearning OR (virtual AND learning) OR (distance AND education) OR (online AND learning) OR (computer-assisted AND instruction) OR (computer assisted AND instruction) OR (digital AND education) OR mobile app* OR virtual reality OR augmented reality OR computer simulation OR smartphone OR tablet OR social media OR Twitter OR Facebook OR Instagram OR TikTok OR WhatsApp)
